# Incidence and risk factors associated with healthcare associated infection of intensive care unit inpatients at Dr. Cipto Mangunkusumo Hospital

**DOI:** 10.1017/ash.2025.151

**Published:** 2025-09-03

**Authors:** Yulia Rosa Saharman, Indah Puspita Sari, R. Fera Ibrahim, Lie Khie Chen, Sharifah Shakinah

**Affiliations:** Department of Microbiology Faculty of Medicine Universitas Indonesia/Dr. Cipto Mangunkusumo Hospital

**Keywords:** Healthcare associated infection, Intensive care unit, risk factor

## Abstract

**Background:** The rate of Healthcare Associated Infection (HAI) in the ICU is five to seven times higher compared to general. The aim of this study was to determine the incidence and risk factors for HAI in the ICU at Dr. Cipto Mangunkusumo hospital. **Methods:** This study use retrospective data, adult patients age ≥ 18 years who were treated in ICU and suspected diagnosis of HAI (including Ventilator associated pneumonia, Catheter associated urinary tract infection, Central line associated bloodstream infection and Surgical site infection) in period from October 2022 – January 2023 were included in this study. We analyze the examination results of each specimen with identification, antibiotic susceptibility test and genomic data using whole genome sequencing. **Results:** There were a total of 160 specimens with 108 positive culture results. The organisms that most commonly cause infections from blood specimens are *Klebsiella pneumoniae* (3/11), *Acinetobacter baumannii* (1/11) and *Pseudomonas aeruginosa* (1/11). For sputum specimens, the causative pathogens obtained included *K. pneumoniae* (23/57), *A. baumannii* (11/57), and *P. aeruginosa* (9/57). Meanwhile, for urine specimen the main bacteria causing infection was *K. pneumoniae* (4/7). In the antibiotic suscpetibility test, the results showed Carbapenem Resistant Organisms (CRO), namely *A. baumannii* 89.5% (17/19), *K. pneumoniae* 76.3% (29/38), *P. aeruginosa* 40% (4/10), and *E. coli* 20% (1/5) with positive ESBL presentation are 15,8% in *K. pneumoniae* and 40% in *E. coli*. **Conclusion:** We found that the most common risk factor for HAI was the use of medical devices. HAI infections that occurred from all the specimens we took were mainly caused by *Klebsiella pneumoniae*. The results of antibiotic resistance are also a matter of note because there are many organism that cause HAI were also Carbapenem-resistant antibiotics with variations in resistance genes (CTX-M, CTX-M-1, SHV, TEM).

